# An Ethno-phenomenological Study of Pregnant Women’s Experiences regarding Household Roles

**DOI:** 10.30476/ijcbnm.2020.84685.1228

**Published:** 2020-10

**Authors:** Fatemeh Erfanian Arghavanian, Abbas Heydari, Mohsen Noghani Dokht Bahmani, Robab Latifnejad Roudsari

**Affiliations:** 1 Nursing and Midwifery Care Research Center, Mashhad University of Medical Sciences, Mashhad, Iran; 2 Department of Midwifery, School of Nursing and Midwifery, Mashhad University of Medical Sciences, Mashhad, Iran; 3 Department of Social Sciences, School of Literature and Humanity, Ferdowsi University of Mashhad, Mashhad, Iran

**Keywords:** Experience, Household, Pregnant women-women’s role

## Abstract

**Background:**

Household labor has been indicated as a feminine role even in the present millennium, in which gender role orientations have been changed. As pregnancy is an important time for studying the division of household labor, this study aimed to discover the meaning of the pregnant women’s experiences of household roles.

**Methods:**

An ethno-phenomenological study, in which van Manen approach to phenomenology was its core and focused ethnographic approach was its supplementary component, was used to conduct this study. 25 pregnant women with maximum variation were recruited via the purposeful sampling during 2016-2017 in Mashhad, Iran. In-depth semi-structured interviews, vignette interviews as well as observations were used for data collection. Six-step van Manen’s descriptive-interpretive phenomenological approach was used for concurrent data collection and analysis. MAXQDA, version 10, was used for data organization.

**Results:**

Data analysis led to the emergence of an overarching theme entitled: “couples’ preservation, keeping up and protection of the household roles”. This was derived from two subthemes including the mother’s efforts to play the household roles and spouse confrontation with the household chores.

**Conclusion:**

The consequence of all endeavors of pregnant women along with their husbands develops the experience of preserving and maintaining the importance of household roles. As pregnancy is an important period for considering division of household responsibilities, it is necessary to design and implement gender sensitive programs to empower pregnant women and their families as well.

## INTRODUCTION

Gender-based social distinctions exist in all human societies and affected the relations between men and women, access to resources, and acceptance of different roles. In general, gender implications affect beliefs, values, behaviors, thoughts, and feelings of individuals and interpersonal interactions as well. ^1 , 2^ Accordingly, gender roles are a set of different acceptable or appropriate expectations regarding the people’s behaviors and beliefs based on their gender, ^3^ and women and men think and behave based on them and manifest their masculinity or femininity. ^4 - 6^

A review of studies indicates that gender and age donate oldest forms of the division of labor. ^7 , 8^ Evidence suggests that women in the traditional societies carry out home-based housework, and out-of-home activities are devoted to men. ^8 , 9^ Therefore, house chores are not a choice for women, and women have no way to refrain from this responsibility. ^7^

In the current century, the gender has a profound impact on the life structure, and changes have been seen in areas like division of labor between men and women. ^10 , 11^ Today, women are not satisfied with old patterns of femininity, and try to play a new social role that had previously belonged to men. ^12 , 13^

During the last decades, characteristics and traditional patterns of Iranian families have been changed and its consequence has been the emergence of a variety of gender related activities. ^14 , 15^ However, despite these changes, women still have the primary responsibility for housework. ^11^ In Iran, definition of feminine role, especially for domestic chores, has still remained in place, and its fulfillment is the main responsibility of women. ^12^

These changes, affect the expectations of pregnant women as well as pregnancy experiences. Certainly, the events and decisions in pregnancy are reflections of the complex effects of such factors as age, gender, norms, and hierarchical values in families. ^16 , 17^ It has been indicated that parenting, compared to other transitions in life, creates the greatest changes in the division of labor, and parents, specifically mothers, find a more traditional gender role. ^18^ On the other hand, parental transition that begins from pregnancy is an important time to examine how the division of labor is done, as it affects the quality of the couples’ relationships and the related experience of this stage. ^19^

Also, gender role has recently been taken into account because of its profound changes. ^20^ However, there is very little information about how the mentioned social conditions affect the division of labor in pregnancy and its meaning for pregnant women. ^19^ It is noteworthy that one of the issues related to pregnancy, beyond medical attention, is division of household labor. ^19 , 21^ There is a limitation of global studies related to the meaning of household role in the experience of pregnant mothers. ^19^ To the best of our knowledge, there is no study in this regard in Iran as well. Thus exploring the experiences of pregnant mothers in relation to this role, particularly, with emphasis on the cultural context of Iran, seems necessary. 

As there is a deficit of knowledge in this regard and due to the dearth of qualitative studies for understanding all aspects of the phenomenon under study, this qualitative research method was used. ^22^ It has been argued that the use of both phenomenological and ethnographic approaches in one study makes it possible to discover the meaning of experience as well as uncover the underlying cultural believes, behaviors and perspectives. ^23^ Therefore, interpretive phenomenology and focused ethnography were used in combination in this study to achieve a broader insight. Having this in mind, this qualitative study was designed to find out the meaning of the pregnant women’s experiences of household roles. 

## MATERIALS AND METHODS

In this qualitative study, an ethno-phenomenological study, in which van Manen approach to phenomenology was its core and focused ethnographic approach was its supplementary component, was used. ^24 , 25^ The two approaches are both exploratory. Also, in both the researcher acts as data collector and uses interviews and a combination of open and structured questions to search the meanings in the experiences of the individuals. ^26^

The study setting included health centers, hospitals, and private maternity clinics of Mashhad, Iran. Participants included 25 Iranian pregnant women selected through purposive sampling during 2016-2017. They had the ability to speak and communicate in Persian, and were willing to participate in the study to share their experiences. Participants who suffered from medical or pregnancy complications that disrupted their daily life were excluded. 

Data collection methods included in-depth semi-structured interviews (n=33) as well as vignette interviews (n=19), observation, field notes and post-interview notes. Eight participants (2, 3.7, 8, 10, 11, 17, and 20) were interviewed twice. The length of interviews was about 39-110 minutes.

Interviews, which were conducted by the first author, initiated with general questions: “How did you feel when you find out that you are pregnant?” and “What was your experience of the pregnancy?” Then, based on the responses and information provided by the participants, the researcher gradually deepened women’s experiences in confrontation with their home duties and the cultural beliefs and behaviors with questions such as “When talking about household duties, what comes to your mind or what’s this experience like?”, “Could you explain your daily activities during your pregnancy?”, “Could you explain your feelings about responsibilities of a pregnant woman in doing housework?”. After each interview, the audio-recorded interviews by a voice recorder were precisely transcribed verbatim. If required, questions were used to further explore the participants’ experiences such as “Can you explain more about this?” or “What do you mean?” were used. 

Accordingly, the researcher conducted vignette interviews with the same participants in this study, in addition to individual interviews. For this purpose, she showed a number of photographs, which were retrieved through an extensive search in electronic databases regarding daily activities, house chores and working outside. It has been tried to portray the modern and traditional gender roles of women and display different scenes of doing work by women or their husbands or in partnership. Then, the researcher asked the women (in the event of a personal experience similar to those photos) to place themselves as the characters in the photo, try to recall their own similar experiences, and explain their feelings towards the photos and discuss with the researcher.

Also if the participants allowed, the researcher went to their homes in the position of “observer as participant” and also conducted interviews and collected the related data. She wrote field notes, based on her field observations of the mothers’ behaviors and actions related to the topic of study. Then, these notes were read out and coded.

To conduct the processes of data collection and analysis, “six-step van Manen’s” interpretive phenomenological approach was used. This approach involves steps including: 1. Getting oriented to the nature of lived experience; 2. Exploring the experience as we live it and not how we conceptualize it; 3. Reflecting about the main themes that reveal the characteristics of the phenomenon; 4. Having the art of writing and rewriting; 5. Maintaining a strong and directional relationship with phenomenon; and 6. Matching the field of research with continuous consideration of components and the whole. ^27^

For the first stage, development of phenomenological questions, clarification of assumptions and pre-understanding of the researcher regarding household role were carried out. In the second stage, the researcher considered a careful and sensitized data collection. In order to obtain the true meaning of the phenomenon and discover the themes, we used thematic analysis using three holistic, selective and detailed approaches. It should be noted that stepping back and considering contribution between the parts and the whole was continuously carried out to make the content more realistic. Also, the researcher tried to write a rich and accurate description of the phenomenon, through the reflection of the participants’ experiences. According to Van Manen, phenomenological data must be directional, strong, rich, and profound. ^27^ For this reason, the researcher carried out the following actions at all stages of the study: having a continuous mental engagement with the research question, maintaining a close relationship and immersion in the data, involving the researcher in the participatory world, having an awareness of all the stages, making an attempt to gain a rich meaning and writing an accurate, in-depth and unique description to formulate a distinctive understanding of the phenomenon under study.

To ensure the validity and rigor of the data, we used four Lincoln and Cuba criteria including credibility, dependability, confirmability, and transferability. ^28 , 29^ The credibility was achieved through prolonged engagement and adequate time allocation to data collection and analysis, member check through getting feedback from participants regarding the extracted data and recruitment of the participants with maximum diversity. Dependability was attained through external review by three experienced researchers. They reviewed and confirmed the extracted data, subthemes and themes. Confirmability was achieved using audit trial. As to the transferability, a detailed description about all aspects of data collection and analysis and participants was presented in the research report. 

The ethical considerations, including obtaining informed consent of the participants, preserving confidentiality of the data, assuring the participants’ right to withdraw from the study at any time, and allowing to use tape recorders, were observed. Also, ethical approval was taken from the Local Research Ethics Committee, Mashhad University of Medical Sciences. Mashhad, Iran. The study was also approved in the research council of Mashhad University of Medical Sciences (Code: 940519).

MAXQDA software 10 was used to organize the data. The recorded interviews were transcribed in the shortest possible time, and all the transcripts were imported into MAXQDA software (version 2010, VERBI Software GmbH, Germany) for data management.

## RESULTS

In this study, 25 pregnant women with a mean age of 32.16±6.55 years participated. The level of their education varied from illiterate up to Ph.D., and their jobs ranged from housekeeper to faculty member (Table 1).

**Table 1:** Demographic characteristics of the participants

Participant	Age(year)	Number of pregnancy	Gestational week	Educational level	Type of employment
1	34	2	34	Bachelor	Accountants
2	33	2(twin)	25	Associate Degree	Housekeeper
3	22	1	22	Associate Degree	Hair stylist
4	30	1	37	Bachelor	Manager and training consultant
5	30	1	36	Bachelor	Housekeeper
6	33	2	24	Ph.D. Student	Instructor
7	37	4	19	Elementary	Housekeeper
8	40	3	17	Illiterate	Cleaner
9	28	1	26	Bachelor Student	Housekeeper
10	36	1	8	Associate Degree	Consultant and Marketer
11	37	1	36	Ph.D.	Assistant professor
12	20	1	12	Bachelor Student	Teacher
13	36	4	28	Bachelor	Housekeeper
14	37	4	34	Associate Degree	housekeeper
15	38	3	40	Ph.D.	Assistant professor and senior manager of the university
16	36	3	39	Elementary	Housekeeper
17	24	1	32	Bachelor	Midwife
18	30	1	34	Master’s degree	Instructor
19	15	1	39	Elementary	Seller
20	30	4	32	Diploma	Tailor
21	39	2	12	Ph.D.	Engineer
22	40	2	14	Bachelor	Housekeeper
23	34	2	39	Ph.D.	Housekeeper
24	27	2	39	Bachelor	Employee
25	38	4	19	Elementary	Housekeeper

Data analysis led to the emergence of an overarching theme titled: “couples’ preservation, keeping up and protection of the household roles”. This theme was derived from two sub- themes and seven sub-sub-themes, which is presented in Table 2.

**Table 2:** Extracted sub-sub-themes, sub-themes and themes

Sub-sub-theme	Sub-theme	Theme
Maintaining the central role of a women in doing house chores	Mother’s efforts to play the household roles	Couples’ preservation, keeping up and protection of the household roles”
Mother’s preference for independent management of life affairs		
Asking others to help in house chores		
Gender tracking in house chores		
Spouse preferences in performing house chores	Spouse confrontation with the household chores	
Spouse’s expectations of woman in playing household role		
The extent of participation of the spouse in house chores		

“Couples’ Preservation, Keeping Up and Protection of the Household Roles”

 The consequence of the couple’s efforts in the face of duties is related to this role, cause and motivations to safeguard this role in a variety of ways. This theme consists of two sub-themes including “the mother’s efforts to play the household roles” and “spouse confrontation with the household chores”.

### 1. Mother’s Efforts in Playing the Household Roles 

This Sub-themes consisted of four sub-sub-themes: “maintaining the central role of a women in doing housework, mother’s preference for independent management of life affairs, seeking help from others in house chores and gender tracking in house chores”. It would seem that mothers put all their concerns in order to be able to maintain their central role in this field. They often represented it in an independent manner. Mothers sought assistance from others in the case of a problem for the realization of this role and importance of its emergence. Truly, mothers tried to preserve their crucial role in house chores. 

### 1. a. Maintaining the Central Role of Women in Doing Housework

The majority of mothers tried to maintain their unique role in doing house chores and complete their efforts in this matter. However, in the lack or reduced ability due to pregnancy, this situation provoked them and led to their dissatisfaction from this situation.

“But the feeling of femininity that you have and you want everything to be perfect like you want your house to be clean, you do your feminine responsibilities, and this leads to a situation that you want to take care of everything and if you can’t deliver it, it makes you so anxious and annoyed. I cried so much that why I couldn’t do my housework. This gives you a bad feeling”. (P11, 36 week)

In such a way, even after all the problems of pregnancy are gone, all the women start doing household promptly because they, as the woman, who are the symbol of life and living, wanted to prove that the life is goes on, with preserving their roles. Some of them state that proving this position increases their self-esteem. 

*“After the second month, you had to admit that you’re becoming a mother. You should live your life beside that. My activities are like before; I do daily routines, cooking, and housework. After that, I’m in a better mood because I’m busy.” (P3, 22 week)*

It is worthwhile to mention that during the interview with this participant (in her mother`s home) and despite her insistence on doing work alone, the researcher noticed that her mother is doing all the work. In the next complementary interview with this participant, the researcher asked about this matter. She answered due to her mother’s obsession; she did not allow her to work in her mother’s home.

The majority of participants believed that it was not necessary to consider pregnant women as disabled people, and they didn’t feel comfortable with these beliefs, attitudes or behaviors, and were reluctant to present such ideas. They acknowledge that pregnancy not only indicate their weakness and reduction of their responsibilities, but also they face a new responsibility.

“It annoys me when they tell me to sit. I tell why I can’t cook. It makes me feel that something has happened to me. I don’t like to lose my activities. I’d like to be like before; just a baby is beside that.” (P24, 39 week)

It seems that pregnancy has made many of women more strong-minded to complete their home responsibilities and has improved feminine roles and even in creative innovative manner.

“Because I used to care so much about cooking; when I got pregnant even though my husband doesn`t force me, but I was encouraged to do very well even in a higher level.” (p5, 36 week)

### 1. b. Mother’s Preference for Independent Management of Life Affairs 

All the women were trying to play this role by not being dependent on others and not disturbing them, and somehow tending to act independently e.

“I didn’t want to be dependent on anyone; I wanted to keep my independence and it was very important to me!” (P4, 37 week)

Almost all of them repeatedly stated that family members were telling them to let them do the chores, but the sense of independence and their desire to maintain it in their lives caused them to do their best.

“Yesterday, my sister told me not to flush the WC and let her do it for me, but I did it myself. I do not like to be a burden fort anyone.” (P9, 26 week)

### 1. c. Asking Others to Help in House Chores

Almost all the mothers were so concerned or worried about doing things when facing pregnancy complications. They would even get help from their husband, children, or family to face the issues and perform housework in a perfect manner.

“My morning sickness was in Ramadan. I could not prepare the meals; I called my mother in law and told her please come and help my husband.” (P5, 36 week)

Some of them stated it was obvious that in the event of pregnancy complications, these changes were temporary, but they were expecting to be understood by their family, because it was important for them to play this role.

“There are some heavy stuff that I can’t lift by myself. I expect my family to help me.” (P7, 19 week)

### 1. d. Gender Tracking in House Chores

Some of the mothers believed that while men and women should not be completely getting away from the role of opposite gender, but they should maintain their specific gender roles. For instance, men and women should fulfill their main masculine and feminine roles at home and these two genders should not be considered the same. The majority of women did not want to delegate all affairs to their husbands, or did not expect to replace the roles of men and women. Some of them stated that in division of duties, it was necessary to pay attention to the delicacies, which were placed in the creation of each sex. 

*“There is a difference between women and men; for example, the expectation from a man should never be as equal as a woman.” (P2, 25 week)*.

During the interview through vignette, a participant after looking at a picture that showed a dad when he was drying his child told: 

”I say no to some things. Some things can’t be understood by men, like the sensitivity of taking care of a child up to age two; fathers can’t really do that, but mothers should do their maternal responsibilities, the ones that are in their expertise; and the father should deal with his familiar chores like dishwashing.” (P1, 34 week)

### 2. Spouse Confrontation with the Household Chores

Men, as quoted by their wives, also showed a wide range of preferences, expectations and practical behaviors about doing housework. 

### 2. a. Spouse Preferences in Performing House Chores

In women’s statements, different preferences of their husbands were reflected regarding house chores. Husbands who didn’t want or prefer to do the housework didn`t cooperate, and their wives were well aware of their preferences; it was obvious for women that they wouldn’t receive empathy from them and put more efforts to do housework. 

“My husband tells me not to touch a thing and he will take care of it, but I end up doing all the work. I know he wouldn’t do it, doesn’t like it and doesn’t matter how much I insist.” (P13, 28 week)

Of course, some spouses, despite the tendency to do housework, wouldn’t do them. It seems that factors beyond their inner senses and preference, like their traditional attitudes regarding doing house chores, discourage them from doing so. 

“He likes to help me; he tells me let him help me but at end he just doesn’t do it.” (P25, 19 week)

The majorities of men tended and preferred to have a clean home, but it wasn’t important for them who do so (mother, family, housekeeper). They believed it wasn`t necessary for pregnant women to do the the chores constantly.

“My husband is very careful about the discipline at home, and although he may not oblige me to work at home, he would prefer to even ask his mom to help me.” (P12, 12 week)

### 2. b. Spouse’s Expectations of Woman to Play the Household Role

The majority of women stated that their partners did not change their expectations for doing things; they showed this even practically to their wives.

“My husband would like me to do the traditional activities like cooking delicious food. He may not even mention it, but he judges by his behavior.” (P3, 22 week)

Although there were husbands who lessened their expectation and even stated that mothers should assign the chores to them and have no concern for doing housework.

“My husband says me: “you don’t tell me all the times that you need my help, you know, he’s requested me: “Please whenever you need help, just let me know, let me know anytime you need” (P8, 17 week)

In this regard, although husbands wanted the mothers to care for their daily routine, they did not oblige them in most cases and did not expect their wives to embark on fulfilling their duties of femininity, especially due to pregnancy problems. However, men welcomed the creativity of their wife in doing housework.

“My husband prefers that I care for house chores like before my pregnancy, but I’m not forced to do anything.” (P3, 22 week)

“Even in cooking, he likes to be modern and be creative at home; in setting the table, he doesn’t like routines.” (P19, 39 week)

### 2.c. The Extent of Participation of the Spouse in House Chores

Experiences of mothers with their spouses’ participation in house chores had a continuum from doing nothing, assist pregnant women , to do everything and involving in all the housework. 

Sometimes. this partnership was through an intermediary (worker or buying dishwasher), and according to the spouse’s opinion, it was the most contribution that he could make. 

“He just bought a dishwasher and told me not to touch dishes; ‘let machine wash it.’ He tells me that I just bought the dishwasher.” (P14, 34 week)

Occasionally, spouses who follow their own preferences towards house chores and have an active role in this area directly ask their mother in law to help her daughter.

“He tells me ‘why did you touch this?’ He would always ask my mother or sister to take care of me when I do something.” (P6, 24 week)

Women also talked about their husbands’ occasional and concise help.

“Last night, he found out my daughter is not well and I was not well too. Then he helped me; sometimes he would help...” (P7, 19 week)

Many participants talked about helping their husbands in housework

“He is part of the housework, and now he’s doing what he can do.” (P6, 24 week)

In some participants, the emergence of male masculinity in participation appeared only in doing heavy work.

“He doesn’t help with housework; he believes the housework is the women’s duty, but when he sees something is heavy or puts pressure on me, he helps me.” (P21, 12 week)

Some participants stated that their spouses were doing all their housework and their home was merely a resting place.

“He really likes to cook and wash at home. I really don’t do anything. He even prepares dinner and sets the table. I only come to eat and he cleans.” (P4, 37 week)

Considering the spouses’ beliefs regarding house chores, mothers told most men did not consider housework to be feminine; they were cooperating and helping. However, the spouses who believed in the femininity of the housework had not a significant cooperation with mothers in housework. 

Women generally stated that the environment in which the spouse has grown uphas a significant impact on the conception of beliefs in this regard. Women stated if their husband experienced constant support from their fathers at home, this would have a significant impact on the development of their gender schema regarding housework.

“The preparing food washing the dishes are done by my spouse’s father, and he does not say this is not a man’s job and a man should never wash the dishes. My husband is like his father know.” (P2, 25 week)

## DISCUSSION

The results of the present study indicated that the consequence of all emotional and practical endeavors of pregnant women along with thoughts and behaviors of their husbands creates the experience of “preservation, keeping up and protection of the household roles” for pregnant women. 

This experience was a continuum of loneliness of pregnant women until their leadership and management in implementation of these duties. Pregnant women in different ways tended to maintain their unity, dominance, and central role in their homes, preferably in an independent manner. When necessary, they draw on the cooperation of others to play this role. In this regard, men confront with house chores with changes and variations in their beliefs and behaviors about housework.

Along with these efforts, mothers tended to preserve a gender segregation rule, not from a domination point of view, but in the context of maintaining the position of couples in the family. They neither want to continue traditional beliefs nor change the role of the spouse. Pregnant women stated that the couples’ collaboration in doing housework and husband’s sensitivity to the needs of their wives bring a more pleasant experience for them.

Studies about pregnancy suggested that changes in the situation of pregnant women can lead to avoidance of home-based duties; thereby leading to adverse reactions ^30^ could affect marital satisfaction during pregnancy. ^31^

In this study, the participants’ experiences with the lack of empathy of the spouse revealed a deep sense of loneliness, while in the case of participation of their spouses, they will experienced satisfaction.

In studies regarding traditional division of housework, it is noted that women are not pleased to do the house chores. Even women who claim to be satisfied with housekeeping, the nature of their housework does not have much reverence, and their usual complaint is loneliness. ^7^

Also, imbalance in division of tasks after the birth of the newborn affects the couples’ performance. ^21^ It has been mentioned that although the parents are more likely to adopt more traditional roles while transition to parenting, this is more true in women, so hours spent by women at home are distinctly higher than men. This leads to a gender gap in division of chores. ^21^

The statements of mothers showed that they had no tendency to diminish their gender role in the family, and they had the responsibility of doing house chores, not because of accepting the traditional role, but to raise their power, leadership and conscientiousness.

In a study on performing domestic duties, there was a good trait for women and neglecting these duties was considered as a negative characteristic for women. ^32^

It has been argued that it is love that encourage women to perform the housework, and women are keen to do it to be pleased. ^33^ Women’s effort in the current study in doing home chores and the importance of maintaining the housekeeping position is in line with this finding.

Another theme in this study was the approach of the spouse to home chores. Stated experiences of the participants showed that the husbands’ beliefs about masculinity or femininity of housework played a significant role in doing those by them. Husbands who believed that housework is the women’s duty avoided doing the chores and vice versa. Researchers believe that cultural beliefs, gender roles and community norms affect the men’s performance and are obstacles to male participation in house chores and raising a child. ^34^

The participants’ experiences revealed that although their husbands did not consider obliging them to do housework, but they emphasized on the importance of doing house chores by women and liked to have clean home. In some cases, men had less expectations from women in doing housework.

In a study, the fathers acknowledged that they were tasked to play active role in house affairs, arguing that reducing the expectation from the pregnant mother would help them to reduce their pressure. ^35^ This finding is consistent with that of the present study. 

However, most of the husbands mentioned that they do not help their wives because they are women’s duties, and their participation may be considered as interference in feminine roles. ^35^ In the present study, some spouses had the same beliefs.

Generally, different studies show that fathers are moving toward adopting new and modern roles although they do not forget their traditional roles. Although the father’s participation in the new affairs, such as house chores, has become even more prominent; ^36 - 39^ nevertheless, this participation is negligible and men assume other important duties for themselves. ^34 , 40^ In Tanzania, although the fathers’ participation has increased, they are still considered as the breadwinner and do the housework is their spouse’s duty. ^41^

In general, evidence suggests that the gender division of labor has existed since the creation of human. ^8^ It should be considered that, in a moral family, its members must behave respectfully with each other and strive to reduce the burden on women through the fair division of housework. ^7^ Especially nowadays, in our society, which is witnessing a change in the social norms of gender-based duties, the pressure of these changes and lack of flexibility of men to accept these changes have caused a double pressure on women. ^2^ As surveys show, despite the changes that have emerged in the orientations and attitudes of societies, housework is considered as a primary task for women, and husbands, despite accepting these notion, have a supplementary role to just help the women. ^2 , 6 , 42 - 44^

In Iran, by analyzing the family behaviorss, it has been found that the general culture of the society is based on helping couples each other, but still doing housework is considered as the main task of women. ^7 , 35^ The findings of this study also confirm this matter. In fact, due to incomparability of pregnancy period to other stages of life ^18^ and the fact that pregnant women are among the most vulnerable groups in the society, ^7 , 45^ it seems that increasing awareness and changing social beliefs can lead to an increased participation of men in house chores. ^35^ It was found that although pregnant women consider doing house chores as their main duty and regarded it as their own central responsibility, but they meticulously play their spousal role to provide constructive interactions with their spouses to get their help and indeed their husbands tend to be supportive during pregnancy as well. ^46 , 47^

Pregnancy is considered as an important time to investigate the division of work in the house, especially if these divisions bring new relationships and communication between the couples. ^6^ Thus, it is necessary to consider the essential policies and training on the division of labor between the couples and family members in pregnancy. ^2^ Then, it is recommended to have a new insight for designing culture and gender based program regarding household roles.

One of the strengths of this study was that it is the first study that thoroughly examines the household role of pregnant women with a gender perspective in the Iranian cultural context. Using vignette is another strength of this study. One of the limitations of this study was the impossibility of living with participants at their homes, although the researcher struggled to have an interview with the participants at their homes. Another limitation was lack of generalizability of the findings due to the nature of qualitative research.

## CONCLUSION

The consequence of all endeavors of pregnant women along with their husbands develops the experience of preserving and maintaining the importance of household roles. Therefore, designing of suitable training programs, tailored to the culture with a special look at gender issues, especially in the Iranian society which is in the path to development, is essential. It is also important to train the families and society to raise their level of knowledge, insight and performance to see the house chores as shared responsibility of husband and wife, particularly during pregnancy. 
